# Analysis risk factors of long-term adverse outcomes and a prediction nomogram for coronary artery disease patients underwent fractional flow reserve

**DOI:** 10.7150/ijms.106807

**Published:** 2025-02-18

**Authors:** Fanqi Li, Jiayi Zhu, Jiabao Zhou, Gaoming Zeng, Yong Zhou, Qiuzhen Lin, Zixi Zhang, Siyuan Tan, Qiming Liu

**Affiliations:** Department of Cardiology, The Second Xiangya Hospital of Central South University, Changsha 410000, China.

**Keywords:** coronary intermediate stenosis, fractional flow reserve, long-term adverse outcomes, nomogram, diabetes mellitus, hyperuricemia

## Abstract

**Background:** The role of fractional flow reserve (FFR) in intermediate lesions has been widely used and recommended by guidelines. However, the long-term outcomes in patient with an intermediate stenosis received FFR have not yet been investigated comprehensively.

**Methods:** We retrospectively included 558 patients underwent both coronary artery angiography (CAG) and FFR. Multivariate logistic regression analysis was employed to identify the independent predictors of major adverse cardiovascular and cerebrovascular events (MACCEs). Additionally, we constructed a prediction nomogram and tested its performance by multiple methods.

**Results:** During a median follow-up of 6.2 years, 87 (15.59%) adverse events were documented. Multivariate logistic regression results revealed that age (OR 1.13, *p*<0.01), diabetes mellitus (OR, 5.87, *p*<0.01), hyperuricemia (OR, 2.91, *p*<0.01) were independently associated with MACCEs. The nomogram consists of age, smoking, hypertension, diabetes mellitus (DM), hyperuricemia, and FFR≤0.8 six factors. The AUC of 3-year, 5-year, 7-year receiver operating characteristic (ROC) curves of training set were 0.697, 0.823, 0.854, and of validation set were 0.845, 0.924, 0.856. The calibration curves and decision curve analysis (DCA) illustrated the ability of the nomogram to predict long-term adverse outcomes and its net benefits in clinical practice.

**Conclusions:** Age, DM, and hyperuricemia were independently associated with long-term adverse outcomes, and the constructed nomogram may be used as a visible tool to predict long-term adverse outcomes for patients underwent FFR.

## Introduction

Percutaneous coronary intervention (PCI) has become the principal treatment for severe coronary artery disease (CAD) and continues to develop by leaps and bounds^1^. Meanwhile, for stable CAD, stent placement is of no benefit if the angiographic stenoses are not responsible for ischemia^2^. FFR is a functional and physiological index used to evaluate coronary blood flow. Exciting developments over the past few years have led to a class IA recommendation by authoritative guidelines^3^. Interventionalists can no longer rely on PCI based on angiography alone. However, the long-term (more than 5 years) outcomes in patient with an intermediate stenosis who received FFR have not yet been investigated comprehensively. A retrospective study at the Mayo Clinic involving over 7,000 individuals revealed that the MACCEs at 7 years was as high as 50.0% in the FFR-guided group^4^. Thus, for patients who have undergone FFR, their long-term prognosis remains unfavorable. In addition, with the popularity of FFR-guided revascularization procedures and the increase in clinical studies related to FFR, it is now evident that the long-term prognostic outcomes of patients with FFR-guided may not be the same in all patient subgroups. A pooled analysis of 879 patients who underwent FFR with FFR >0.75 concluded that gender differences in 5-year long-term prognosis, with women having a better outcome^5^. Our previous work also showed that high uric acid levels were related to increased MACCEs among patients underwent FFR^6^. To summarize, the long-term outcomes of patients who received FFR remains to be improved and varies across subgroups. Therefore, we retrospectively collected medical information and followed up with these patients in an attempt to identify risk contributors to MACCEs.

The long-term prognosis of risk factors like smoking, hypertension, and DM on cardiovascular events has been extensively studied and incorporated into treatment guidelines for managing CAD^2^. However, the effect of these traditional cardiovascular risk factors on patients with an intermediate stenosis received FFR were uncomprehensive. Nomogram is a visual model that integrates multiple predictors based on a regression equation. Hence, we aim to construct a nomogram to predict long-term clinical prognosis for CAD patients who received FFR.

## Methods

### Study population

The study was a retrospective cohort design implemented at Beijing Anzhen Hospital, affiliated with Capital Medical University. This study consecutively involved 1198 individuals who underwent CAG and FFR from October 2014 to December 2019. The exclusion criteria have been detailed in our prior research^7^. Briefly, it included history of revascularization, acute myocardial infarction, chronic total occlusion, glomerular filtration rate < 60 ml/min, multivessel disease, and absence of information. Ultimately, a total of 558 patients were enrolled. This study design approved by Beijing Anzhen Hospital.

### Procedures and treatment

All interventional procedures are operated by qualified interventionalists in adherence to guidelines. Should two experienced operators classify the CAG findings as indicating an intermediate lesion, an FFR assessment will carry on upon obtaining informed consent. During FFR measurements, adenosine triphosphate was administered through the median elbow vein via a high-flow intravenous infusion pump at a continuous constant rate to obtain a state of maximal coronary artery congestion. Afterwards, FFR-guided PCI was performed, and stenting was considered only if the FFR≤0.8. All patients received standardized treatment, and no major surgical complications occurred during hospitalization.

### Data collection and definition

All patient data were retrieved from the hospital's information system, encompassing demographic details, laboratory results, prescribed medications, and other relevant clinical information. By October 2023, follow-up assessments for all patients were completed via the hospital's outpatient system or through telephone contact. The MACCEs included repeat revascularization, nonfatal stroke, nonfatal myocardial infarction, and all-cause death.

The diagnosis of hypertension and DM is based primarily on medical history and the oral hypoglycemic and antihypertensive medications the patient is taking. For those patients who have not yet been definitively diagnosed, the diagnosis is based on fasting blood glucose results and sphygmomanometer measurements. The diagnosis of hyperlipidemia is based on the measurement of cholesterol and triglycerides levels^8^. Smoking refers to having smoked for a continuous period of more than six months. Definition of hyperuricemia adopted a sex-based threshold approach, 360 mmol/L for women and 420 mmol/L for men^9^.

### Outcomes

The median duration of follow-up for all patients in the study was 74 months. The primary endpoint was a composite of MACCEs. A total of 87 MACCE events were recorded throughout the follow-up period.

### Statistical analysis

Continuous variables are expressed as either the mean ± standard deviation or the median (P25, P75), depending on the normality of their distribution. Group comparisons were conducted using Student's t-test or the Wilcoxon test. Categorical variables are displayed as frequencies and percentages, analyzed with the chi-square test or Fisher's exact test where appropriate. All participants were randomly assigned to the training set and validation set at the ratio of 7:3. Most factors are not statistically different between the training and validation sets (*p*>0.05). Univariate and multivariate Cox analysis were used to identify independent factors of MACCEs in the training set. Based on statistical and clinical significance, we choose age, smoking, hypertension, DM, hyperuricemia, and FFR≤0.8; six feasible factors for the construction of the nomogram. Six selected factors were used to construct nomogram in the training set. AUCs, C-indices, and calibration curves were calculated to evaluate the predictive efficacy of the nomogram. DCA was plotted to assess the net benefits of nomogram in clinical practice. SPSS 26.0 were used for data analysis (IBM Corporation, Armonk, NY, USA). The construction and evaluation of nomogram were performed with R software using the “regplot” “time ROC” “survival ROC” and “pec” packages. For all statistical calculations, *p* values < 0.05 were considered statistically significant.

## Results

**Figure 1:**
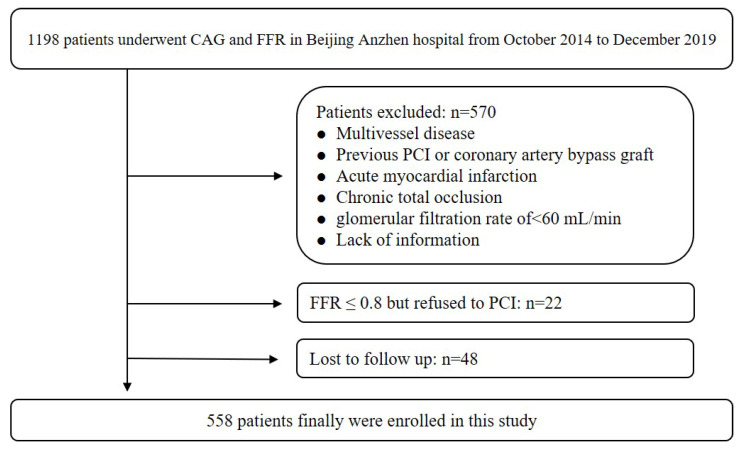
Population flow chart of enrolled patients.

A total of 558 patients were ultimately enrolled, and the recruitment pathway is illustrated in Figure 1. The average age of all patients was 57.76±8.95 years, with 375 (67.20%) being men. Over a median follow-up of 74 months, a total of 87 (15.59%) MACCEs were recorded. The average age in No-MACCE group was 56.88±8.70, and was 64.83±7.90 in the MACCE group. The incidence of DM was 22.51% in the No-MACCE group and 59.77% in the MACCE group. The prevalence of hyperuricemia in No-MACCE group was 15.29%, and was 35.63% in MACCE group.

### Baseline characteristics

The baseline information categorized by the incidence of MACCE were presented in Table 1. Patients suffered from MACCE were older and more likely to have hypertension, DM, hyperuricemia, elevated uric acid (UA), higher CRP levels, and lower FFR value. Additionally, they had a higher incidence of PCI compared to No-MACCE category (*p*<0.05).

### Identify valuable factors and nomogram development

**Figure 2:**
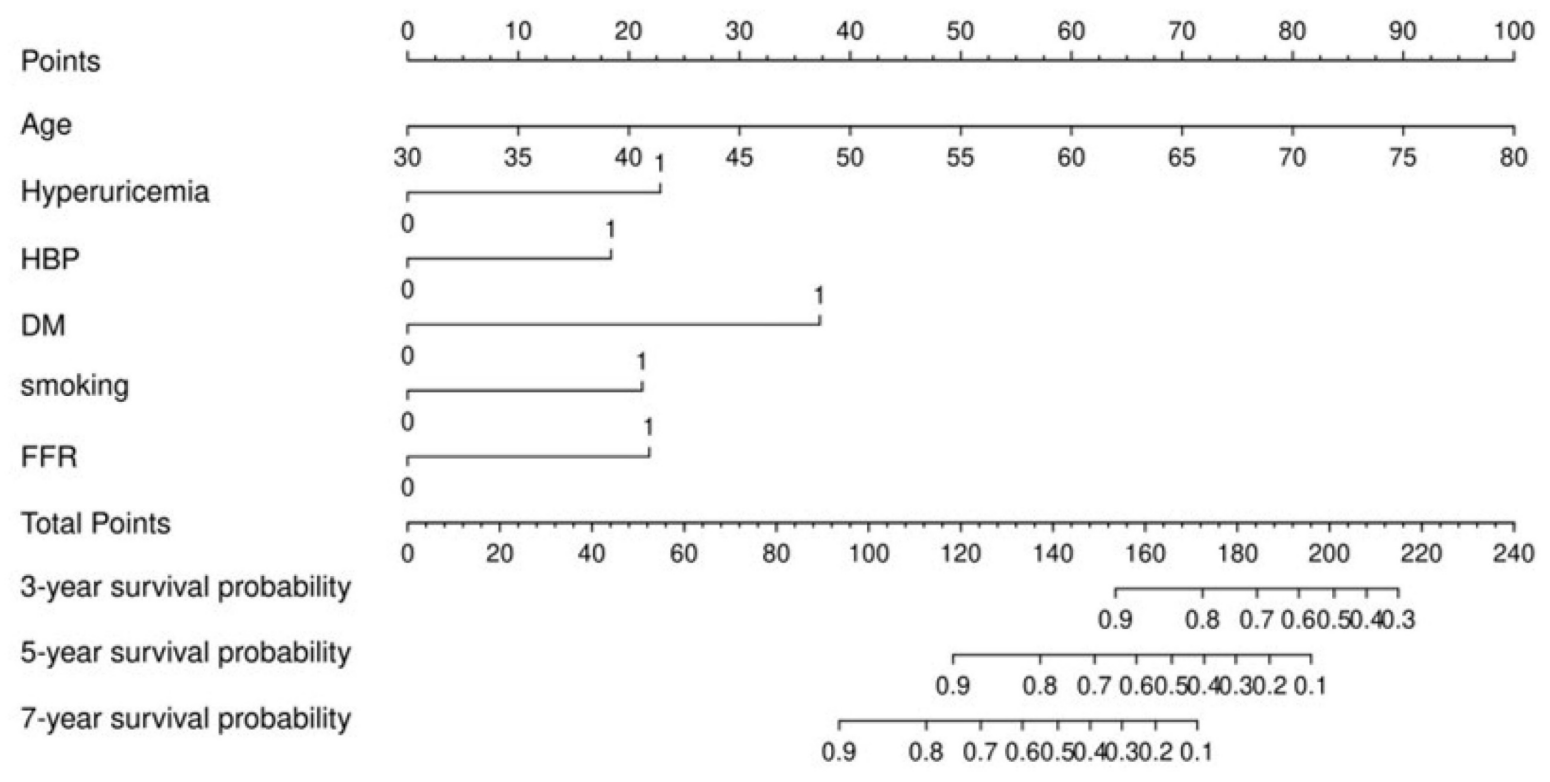
Nomogram for predicting 3-year, 5-year, and 7-year survival probability in patients underwent FFR measurement with an intermediate stenosis.

In the training set, Cox regression analysis was performed to screen factors for construction nomogram. Univariate Cox regression analysis showed that the factors of age, hypertension, DM, hyperuricemia, UA, FFR≤0.8, and FFR were statistically significant (*p*<0.05) (Table 2). Based on statistical and clinical significance, we choose age, male, BMI, hypertension, dyslipidemia, DM, smoking, hyperuricemia, LDL-C, and FFR≤0.8 for the multivariate Cox analysis (Table 2). From the multivariate Cox analysis results, we finally identify age, smoking, hypertension, DM, hyperuricemia, and FFR≤0.8 six factors for the construction of nomogram. The nomogram was shown in Figure 2.

### Nomogram validation

**Figure 3:**
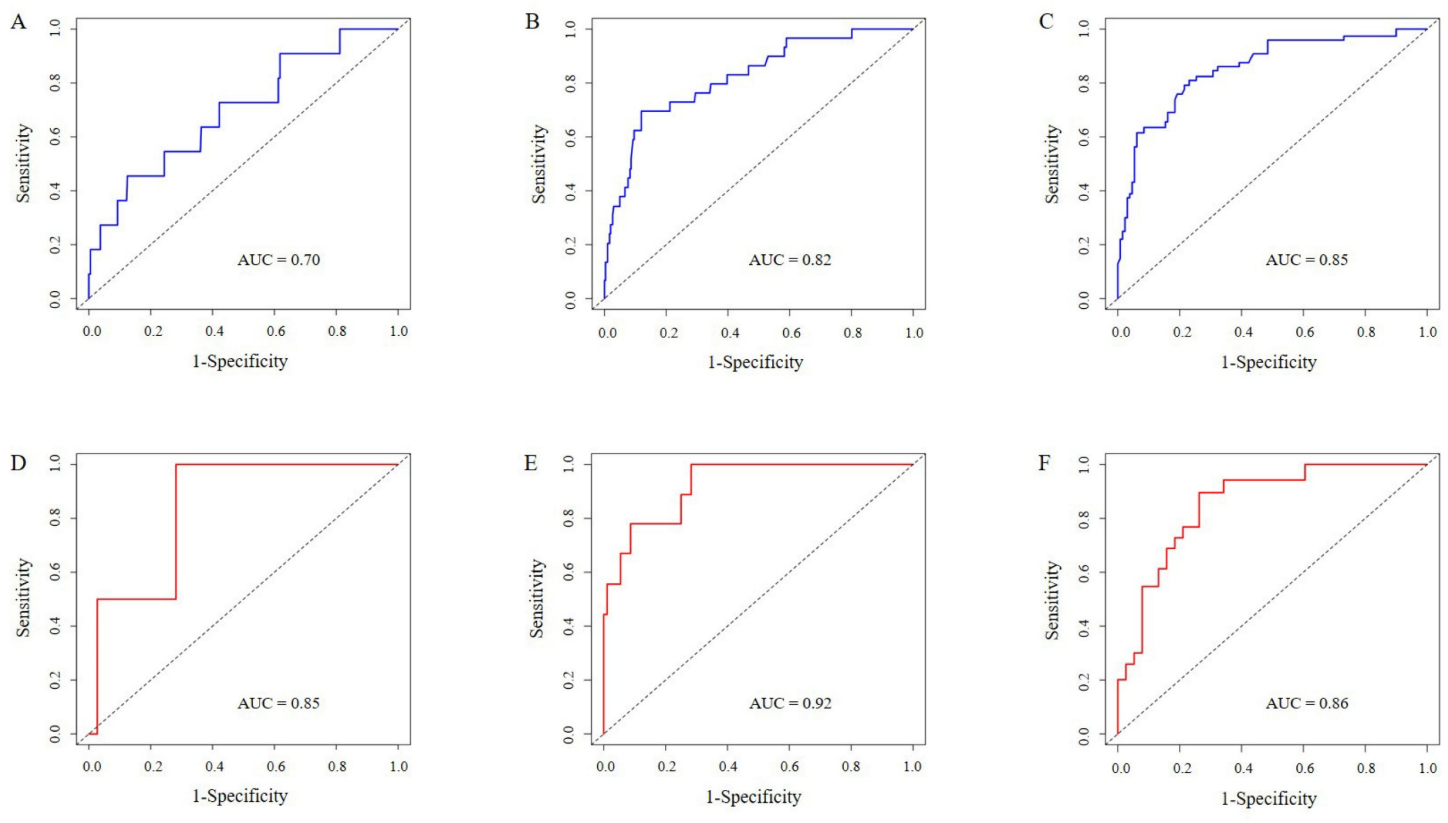
ROC curves for testing the effect of nomogram. 3-year, 5-year and 7-year survival ROC curves in the training set (3A, B, C). 3-year, 5-year and 7-year survival ROC curves in the validation set (3D, E, F).

**Figure 4:**
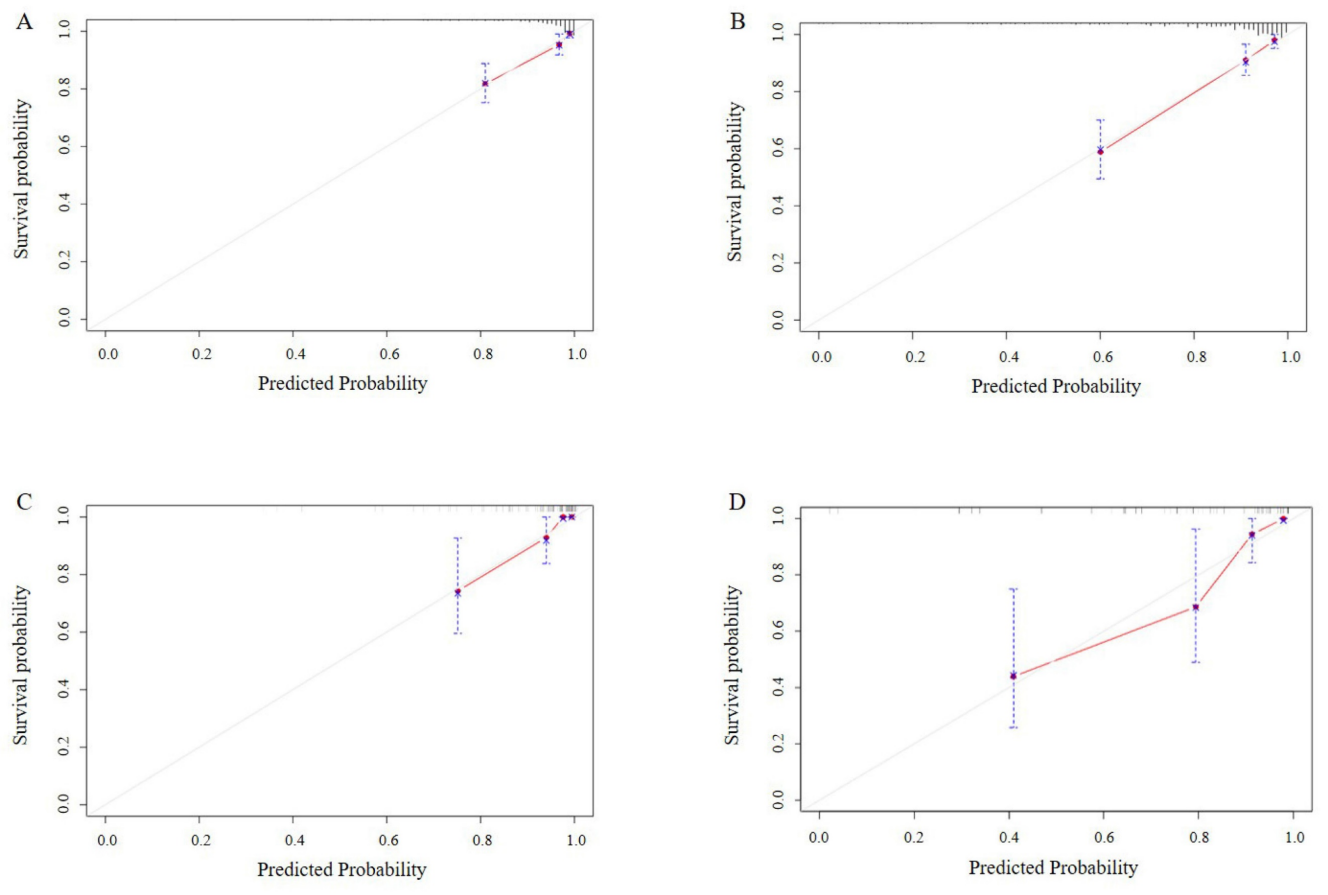
Calibration curves for testing the effect of nomogram. 5-year and 7-year survival calibration curves in the training set (4A, B). 5-year and 7-year survival calibration curves in the validation set (4C, D).

**Figure 5:**
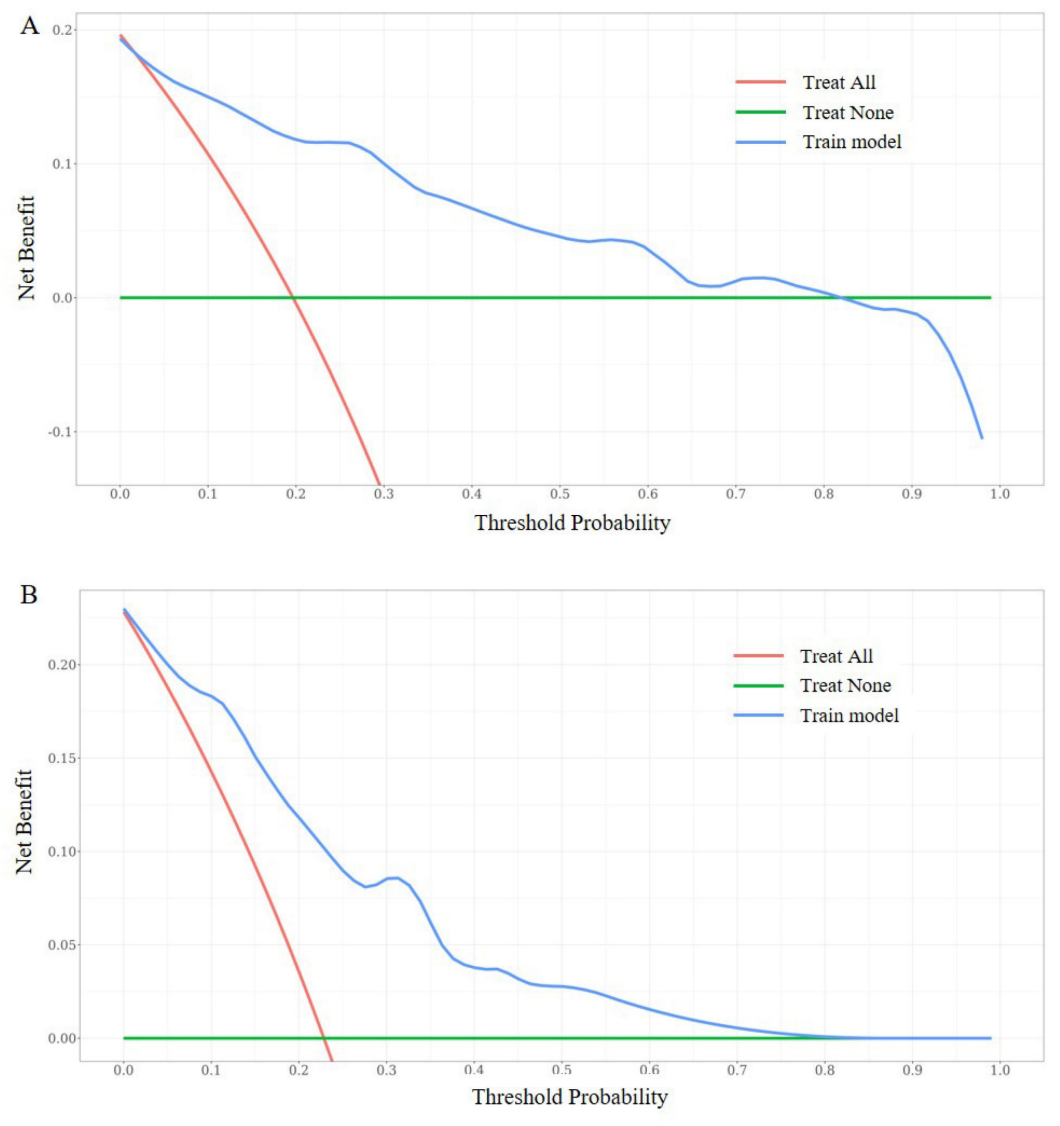
DCA curves of the nomogram. DCA curves at 7-year in the training set (5A). DCA curves at 7-year in the validation set (5B).

In our study, multiple methods were employed to evaluate the predictive performance of the model, including the C-index, ROC curve, calibration curves, and DCAs. In the training set, the C-index was 0.807 (95% CI 0.749-0.866). The AUC of 3-year, 5-year, and 7-year AUCs were 0.697 (95% CI 0.536-0.859), 0.823 (95% CI 0.741-0.904), and 0.854 (95% CI 0.790-0.918) (Figure 3A, B, C). In the validation set, the C-index was 0.832(95% CI 0.754-0.911). The AUC of 3-year, 5-year, and 7-year AUCs were 0.845 (95% CI 0.662-1.029), 0.924 (95% CI 0.850-0.999), and 0.856 (95% CI 0.758-0.954) (Figure 3D, E, F). The calibration curves showed an ideal agreement for 5-year and 7-year survival prediction in the training and the validation categories (Figure 4). The DCA illustrated 7-year net benefits of nomogram in clinical practice in both training and validation categories (Figure 5).

## Discussion

In this study, we investigated the risk factors that compromise the prognosis of patients underwent FFR for coronary intermediate stenosis and concluded the following two main findings. First, age, DM, and hyperuricemia were independently linked to long-term adverse outcomes for patients with coronary intermediate stenosis. Second, the constructed nomogram may be used as a visible tool to predict long-term adverse outcomes for CAD patients with an intermediate stenosis.

The primary aim of coronary revascularization is to alleviate ischemia and restore blood flow in the coronary arteries. Treating coronary stenosis with stenting enhances exercise tolerance, decreases the need for anti-ischemic drugs, and improves survival rates in patients experiencing ST-elevation myocardial infarction. However, for those with stable coronary artery disease, stent placement offers no advantage if the angiographic stenoses are not causing ischemia^2^. The reasoning behind physiologic lesion assessment is straightforward: for lesions with moderate severity, angiography alone is insufficient to guide revascularization. Coronary angiography provides two-dimensional images, representing a silhouette of the three-dimensional vascular lumen from a specific angle. It does not directly reveal atherosclerosis, which affects the vessel wall, but instead creates a “shadow” that lacks detailed intraluminal information necessary for characterizing plaques. Additionally, angiographic limitations, such as contrast streaming, branch overlap, vessel foreshortening, calcifications, and issues with ostial origins, can make interpreting certain luminal narrowings challenging^10^. FFR provided a new perspective by evaluating stenosis based on its physiological impact on blood flow. After decades of development, FFR has gradually become the recognized gold standard for functional assessment indexes^11^, ^12^, ^13^. However, despite the application of FFR, the long-term prognosis in these patients may fall short of expectations. A retrospective study at the Mayo Clinic involving over seven thousand individuals demonstrated that the 7-year MACCE rate in the FFR-guided group reached 50.0%, though lower than in the CAG-guided^4^. Another study reported a 5-year MACCE rate of 38.2%. Additionally, among male patients who deferred revascularization with an FFR > 0.75, the 5-year adverse event rate was 10.5%. Thus, for patients who have undergone FFR, their long-term prognosis remains unfavorable. Meanwhile, the comprehensive evaluation of long-term outcomes (exceeding 5 years) in patients who have undergone FFR assessment remains lacking in the current literature. Therefore, it is essential to investigate the long-term prognosis in these patients and implement proactive interventions. Our results proved that age, DM, and hyperuricemia were independently associated with long-term MACCEs for patients underwent FFR measurement with coronary intermediate stenosis.

Elderly patients are at high prevalence and high risk for cardiovascular disease. Aging cardiac cells have been proven to be associated with atherosclerosis^14^. The progress of cardiac cellular senescence exacerbates the severity of cardiac pathologies by the mechanism of age-related telomere shortening, metabolic dysfunction, oxidative stress, and other stressors^15^. Numerous clinical studies have also confirmed that elderly patients with cardiovascular disease have a worse prognosis^16^, ^17^. Our findings similarly suggest that older patients tend to have a worse prognosis.

In recent years, numerous large-scale population studies in China have identified a significant comorbidity between cardiovascular disease and glucose metabolism abnormalities, with this association further compounding cardiovascular risk^18^, ^19^. Findings from the China Cardiovascular Disease Quality Improvement Project reveal that the prevalence of diabetes in acute coronary syndrome patients reaches 37.6%, with a prevalence as high as 26.9% among those under 45 years. Additionally, ACS patients with concurrent diabetes face a 1.5-fold higher risk of MACCEs and a twofold elevated risk of all-cause mortality^20^. Our results demonstrated patients with DM have a nearly five times higher risk of MACCE than non-diabetics.

Elevated serum uric acid levels, in addition to causing gout, are also associated with the onset and progression of cardiovascular disease. Under normal physiological conditions, serum uric acid saturates at 420 µmol/L. When levels exceed this threshold, monosodium urate crystals may precipitate, adhering to blood vessels and recruiting macrophages and neutrophils. This interaction induces the release of pro-inflammatory cytokines, matrix metalloproteinase-9, and hydrolases, resulting in acute and chronic inflammatory injury to the cardiovascular system^21^. Our study revealed that hyperuricemia independently increased incidence of MACCEs by nearly twofold in patients undergoing FFR.

The nomogram is a visual model that integrates multiple predictors based on a regression equation. This novel statistical approach has been widely used in cardiovascular disease research^22^, ^23^. In our study, we first constructed a nomogram based on FFR for patients with an intermediate coronary stenosis. The factors selected for the construction of nomogram mainly based on statistical and clinical significance. An interesting phenomenon is that FFR≤0.8, a strong determinant for prognosis, did not show statistical significance in our Cox regression model. The reasons for this may be that (1) the participants we enrolled were all single-vessel disease of intermediate stenosis, different from previous studies containing more severe conditions such as multivessel disease and previous revascularization^24^, ^25^; (2) up to 6 years of median follow-up time, the role of DM, age, and hyperuricemia may attenuate the effect of FFR in this lengthy process; (3) the insufficient sample size in the MACCE group. As we all know, statistics are only an aiding technology tool in medical research and clinical significance cannot be neglected^26^. Therefore, we incorporated FFR≤0.8, smoking, and hypertension as valuable factors to construct nomogram, even though they were not statistically significant. As a result, the nomogram constructed from these six factors demonstrated satisfactory predictive efficacy after being tested by multiple methods.

### Limitations

This study has several limitations. First, as a retrospective, single-center study, it may be subject to selection bias. Second, since we included only individuals with intermediate coronary stenosis in a single vessel, the findings need further validation in patients with multivessel disease and other clinical conditions.

## Conclusion

Age, DM, and hyperuricemia were independently associated with long-term adverse outcomes for CAD patients with an intermediate stenosis. The constructed nomogram based on FFR in our study may be used as a visible tool to provide long-term prognosis information for patients undergone FFR assessment.

## Figures and Tables

**Table 1:** Baseline characteristics of patients

	Total (n=558)	No-MACCE (n=471)	MACCE (n=87)	*p* value
Age (y)	57.76 ± 8.95	56.88 ± 8.70	64.83 ± 7.90	<.001
Sex, male	375 (67.20)	319 (67.73)	56 (64.37)	0.540
BMI, kg/m^2^	25.58 ± 3.03	25.62 ± 3.07	25.26 ± 2.71	0.440
Risk factors, n (%)				
Hypertension	270 (48.39)	218 (46.28)	57 (65.52)	0.011
Dyslipidemia	318 (56.99)	272 (57.75)	44 (50.57)	0.383
Diabetes mellitus	149 (26.70)	106 (22.51)	52 (59.77)	<.001
Smoking	188 (33.69)	157 (33.33)	31 (35.63)	0.698
Hyperuricemia	98 (17.56)	72 (15.29)	31 (35.63)	<.001
Laboratory results				
LDL-C, mmol/L	2.31 (1.76 - 2.84)	2.32 (1.75 - 2.85)	2.31 (1.86 - 2.62)	0.770
HDL-C, mmol/L	1.09 (0.93 - 1.30)	1.09 (0.93 - 1.31)	1.09 (1.00 - 1.22)	0.920
TC, mmol/L	3.99 (3.30 - 4.74)	4.00 (3.30 - 4.74)	3.94 (3.30 - 4.45)	0.891
TG, mmol/L	1.37 (0.95 - 1.89)	1.37 (0.95 - 1.90)	1.38 (0.96 - 1.73)	0.605
Uric acid, μmol/L	329 (295 - 384)	326 (291- 379)	361 (334 - 420)	<.001
Cr, μmol/L	66.4 (59.9 - 75.9)	66.3 (59.4 - 75.9)	68.9 (61.8 - 75.7)	0.513
TP, g/L	69.4 (65.0 - 73.2)	69.4 (65.0 - 73.2)	69.4 (65.9 - 73.0)	0.951
CRP, mg/L	0.90 (0.45 - 1.96)	0.87 (0.44 - 1.92)	1.14 (0.74 - 2.43)	0.035
LVEF, %	65.05 ± 5.28	65.13 ± 5.22	65.15 ± 7.04	0.98
Procedure characteristics				
Target vessel territory, n (%)				
LM	19 (3.41)	17 (3.61)	2 (2.30)	0.699
LAD	432 (77.42)	362 (76.86)	71 (81.61)	0.404
LCX	29 (5.20)	26 (5.52)	3 (3.45)	0.695
RCA	78 (13.98)	66 (14.01)	12 (13.79)	0.876
PCI, n (%)	193 (40.29)	156 (37.23)	37 (61.67)	<.001
FFR	82.0 (75.8 - 88.0)	83.0 (76.0 - 88.0)	79.0 (72.5 - 84.0)	0.01
Stent number	1.0 (1.0, 1.0)	1.0 (1.0, 1.0)	1.0 (1.0, 1.0)	0.679
Mean stent diameter, mm	3.16 ± 0.39	3.15 ± 0.38	3.19 ± 0.46	0.772
Total stent length, mm	28.62 ± 12.46	29.67 ± 16.23	28.54 ± 12.18	0.795
Medications at discharge, n (%)				
Aspirin	544 (97.49)	458 (97.24)	87 (100)	0.376
Statin	473 (84.77)	398 (84.50)	77 (88.51)	0.436
β-Blocker	313 (56.09)	268 (56.90)	45 (51.72)	0.453
ACEI/ARB	240 (43.01)	207 (43.95)	32 (36.78)	0.289

MACCE, major adverse cardiovascular and cerebrovascular events; BMI, body mass index; LDL-C, low density lipoprotein cholesterol; HDL-C, high density lipoprotein cholesterol; TC, total cholesterol; TG, triglyceride; Cr, creatinine; TP, total protein; CRP, C reactive protein; LVEF, left ventricular ejection fraction; LM, left main coronary artery; LAD, left anterior descending; LCX, left circumflex; RCA, right coronary artery; PCI, percutaneous coronary intervention; FFR, fractional flow reserve; ACEI/ARB, angiotensin converting enzyme inhibitors/angiotensin receptor blockers.

**Table 2:** Univariate and multivariate analysis

	Univariate analysis		Multivariate analysis	
	OR (95% CI)	*p* value	OR (95% CI)	*p* value
Age (y)	1.12 (1.08 - 1.17)	<0.01	1.13(1.08-1.18)	<0.01
Sex, male	0.71 (0.40-1.28)	0.26	0.99(0.51-1.93)	0.98
BMI, kg/m^2^	0.96(0.88-1.06)	0.45	0.98(0.88-1.09)	0.72
Hypertension	2.37(1.29-4.33)	0.01	1.50(0.78-2.86)	0.22
Dyslipidemia	0.94(0.53-1.66)	0.82	0.80(0.43-1.47)	0.47
Diabetes mellitus	4.39(2.45-7.87)	<.001	5.87(3.09-11.13)	<0.01
Smoking	0.99(0.55-1.80)	0.98	1.58(0.81-3.06)	0.18
Hyperuricemia	2.79(1.54-5.06)	<0.01	2.91(1.51-5.63)	<0.01
LDL-C, mmol/L	0.91(0.63-1.32)	0.63	0.91(0.58-1.41)	0.66
HDL-C, mmol/L	0.84(0.30-2.35)	0.74	-	-
TC, mmol/L	0.95(0.72-1.26)	0.72	-	-
TG, mmol/L	0.82(0.57-1.17)	0.27	-	-
Uric acid, μmol/L	1.01(1.00-1.01)	<0.01	-	-
Cr, μmol/L	1.00(0.98-1.02)	0.93	-	-
TP, g/L	1.01(0.96-1.06)	0.84	-	-
CRP, mg/L	1.03(0.96-1.10)	0.44	-	-
LVEF, %	1.00(0.95-1.06)	0.97	-	-
FFR≤0.8	1.85(1.04-3.28)	0.04	1.65(0.90-3.01)	0.11
FFR	0.96(0.93-0.98)	<0.01	-	-

BMI, body mass index; LDL-C, low density lipoprotein cholesterol; HDL-C, high density lipoprotein cholesterol; TC, total cholesterol; TG, triglyceride; Cr, creatinine; TP, total protein; CRP, C reactive protein; LVEF, left ventricular ejection fraction; FFR, fractional flow reserve.
